# School-Based Interventions Targeting Nutrition and Physical Activity, and Body Weight Status of African Children: A Systematic Review

**DOI:** 10.3390/nu12010095

**Published:** 2019-12-30

**Authors:** Theodosia Adom, Anniza De Villiers, Thandi Puoane, André Pascal Kengne

**Affiliations:** 1 School of Public Health, Faculty of Community and Health Sciences, University of Western Cape, Cape Town 7535, South Africa; tpuoane@uwc.ac.za; 2 Nutrition Research Centre, Radiological and Medical Sciences Research Institute, Ghana Atomic Energy Commission, Accra LG 80, Ghana; 3 Division of Research Capacity Development, South African Medical Research Council, Cape Town 7505, South Africa; Anniza.DeVilliers@mrc.ac.za; 4 Non-communicable Disease Research Unit, South African Medical Research Council, Cape Town 7505, South Africa; Andre.Kengne@mrc.ac.za

**Keywords:** school-based interventions, physical activity, diet, energy balance-related behaviours, overweight/obesity, effectiveness

## Abstract

Background: Overweight/obesity is an emerging health concern among African children. The aim of this study was to summarise available evidence from school-based interventions that focused on improving nutrition and physical activity knowledge, attitude, and behaviours, and weight status of children aged 6–15 years in the African context. Methods: Multiple databases were searched for studies evaluating school-based interventions of African origin that involved diet alone, physical activity alone, or multicomponent interventions, for at least 12 weeks in duration, reporting changes in either diet, physical activity, or body composition, and published between 1 January 2000 and 31 December 2018. No language restrictions were applied. Relevant data from eligible studies were extracted. Narrative synthesis was used to analyse and describe the data. Results: This systematic review included nine interventions comprising 10 studies. Studies were conducted among 9957 children and adolescents in two African countries, namely South Africa and Tunisia, and were generally of low methodological quality. The sample size at baseline ranged from 28 to 4003 participants. Two interventions reported enrolling children from both urban and rural areas. The majority of the study participants were elementary or primary school children and adolescents in grades 4 to 6. Participants were between the ages of 12.4 and 13.5 years. All but one intervention targeted children of both sexes. Four studies were described as randomised control trials, while five were pre- and post-test quasi-experiments. Except for one study that involved the community as a secondary setting, all were primarily school-based studies. The duration of the interventions ranged from four months to three years. The interventions focused largely on weight-related behaviours, while a few targeted weight status. The results of the effectiveness of these interventions were inconsistent: three of five studies that evaluated weight status (body mass index (BMI), BMI *z*-score, overweight/obesity prevalence), three of six studies that reported physical activity outcomes (number of sports activities, and physical activity duration ≥ 30 min for at least six days/week), and four of six reporting on nutrition-related outcomes (number meeting fruit and vegetable intake ≥ 5 times/day) found beneficial effects of the interventions. Conclusion: Given the dearth of studies and the inconsistent results, definite conclusions about the overall effectiveness and evidence could not be made. Nonetheless, this study has identified research gaps in the childhood obesity literature in Africa and strengthened the need for further studies, the findings of which would contribute valuable data and inform policy.

## 1. Introduction

Overweight and obesity are major public health threats worldwide [1]. The prevalence of childhood overweight increased by almost 50% between 2000 and 2015 [2]. In 2016, nearly 41 million children under 5 years were overweight or obese worldwide—of which, 49% and 24% lived in Asia and Africa [2]. Further, we recently estimated 9.4% overweight and 5.0% obesity prevalence among African school children [3].

The consequences of childhood obesity, which may be apparent even early in life, include increased risk for cardiovascular diseases [4] from dyslipidaemia, high blood pressure, and dysglycaemia. Documented evidence form Africa suggests positive associations of markers of metabolic syndrome with increased body mass index, body fat, overweight or obesity [5,6,7,8,9,10]. For instance, in a systematic review and meta-analysis of studies involving children and adolescents in Africa, Noubiap and colleagues [5] reported that elevated blood pressure was associated with body mass index. The blood pressure was six times higher in children who were obese relative to children with normal weight. Among Tunisian [8,9,10] and South African children [11,12], the prevalence of metabolic syndrome was higher in children and adolescents who were overweight/obese. Psychological issues such as stigmatisation and low self-esteem have similarly been reported [4]. Additionally, childhood obesity is a risk factor for adult obesity, and cardiometabolic morbidity [13], highlighting the importance of early prevention.

Given the multifactorial nature of overweight and obesity, there is the need for a multi-disciplinary approach that focuses on the diverse environments in which children live for successful interventions. The school setting has been identified as ideal for health promotion interventions, since children spend a significant amount of time in schools and are exposed to supportive environments such as school health policies, nutrition education and support, physical education, and physical activity (PA) during school hours. Despite these, the evidence from systematic reviews and meta-analyses on the effectiveness of school-based programmes have been mixed [14,15,16,17,18,19]. Moreover, the evidence is mostly from high- to middle-income countries. One systematic review [16] of studies from low- to middle-income countries concluded that overall, school-based programmes are promising in improving behavioural determinants of unhealthy body weight. Only one African study was included in that review, making the generalisation of their findings to African countries a challenge. The objective of the present systematic review was therefore to characterise and summarise available evidence from school-based interventions that focused on improving nutrition and physical activity knowledge, attitude, and behaviours, and weight status of learners aged 6–15 years in the African context.

## 2. Materials and Methods

### 2.1. Inclusion Criteria, Data Sources and Selection of Relevant Studies

The protocol for this systematic review has been previously described [20] and follows the Preferred reporting items for systematic review and meta-analysis (PRISMA) guidelines [21] (online supplementary material Table S1) with PROSPERO registration no. CRD42016041614. To be eligible for inclusion, studies had to be: conducted in school children aged 6–15 years, or presenting data specific for the subgroup of participants within the specified age range, of African origin and residing in African countries; primary research that evaluated dietary interventions only, PA interventions only, or combined dietary and PA interventions for at least 12 weeks in duration; reported changes in diet and PA knowledge, attitude and self-efficacy, increased participation in PA, increased intake of fruits and vegetables, decreased consumption of high-fat diets and sugar-sweetened beverages, changes in body weight, body mass index (BMI) or BMI z-score and reporting baseline and post-intervention measurements; focused primarily on the school setting; prevention and treatment that used a controlled or no control study design, with or without randomisation; published and unpublished studies between 1 January 2000 and 31 December 2018. Post-2000 studies were selected because, hitherto, the focus of research had been on undernutrition in African children; no language limitations were applied. For studies that evaluated multiple outcomes of the same intervention, these are treated as separate studies or the most comprehensive and recent report is included. Studies were excluded if they were clinic-based studies or had no school-based components; conducted among school children with eating disorders, critical illnesses or chronic conditions; in African populations but residing outside the continent.

A comprehensive search of MEDLINE (PubMed), MEDLINE (EBSCOHost), CINAHL (EBSCOHost), Academic Search Complete (EBSCOHost) and African Journals Online (AJOL) was conducted to identify potentially eligible studies. Key search terms relating to population (learners, ‘school children’, ‘school-aged children’, ‘school going children’); interventions (diet-related, PA-related, school environment-related); geographical settings (African search filter [22]); and outcomes (changes in nutrition and PA knowledge, attitude, intention and self-efficacy, and behaviours, changes in body weight, percent body fat, BMI or BMI z-score) were used. The search terms were modified for each database. The search strategy for the PubMed database can be found in the online supplementary material Table S2. The reference lists of identified studies were manually checked for other relevant studies and key specialists in the field were contacted for any unpublished study. References were exported and duplicates removed using Endnote citation manager. The titles and abstracts of potentially relevant articles were independently screened by two reviewers for eligibility. Full-text copies of articles that met the eligibility criteria were obtained and assessed by two independent reviewers for inclusion in the review. Any disagreement about the eligibility was resolved through discussion.

### 2.2. Data Extraction

This was performed by one reviewer using a piloted data sheet that was purposely designed for the study and discussed by two reviewers. The following were extracted: study details (author, year of publication, and country of study); study design; study population (sample size, age, and gender); intervention characteristics (type, content, duration of study, follow-up time points, drop-outs, mode of delivery, and intervention provider); settings; outcome (primary outcomes: changes in body weight, percent body fat, BMI or BMI z-score; changes in intake of fruit and vegetables, and sugar-sweetened beverages, increased participation in PA and physical fitness; other relevant outcomes: changes in nutrition/dietary and PA knowledge, attitude, intention and self-efficacy); intervention effects (as reported by the authors) and theoretical basis of intervention. The corresponding author of one study was contacted for additional information but there was no response. The study was included in the review despite the unavailability of complete data because of its relevance.

### 2.3. Quality Assessment

The “Effective Public Health Practice Project quality assessment tool for quantitative studies” [23] was used to evaluate the methodological quality of the included studies. The studies were rated based on six components, namely, selection bias, study design, confounders, blinding, data collection methods, withdrawals, and drop-outs. For each study, the six components were rated as weak, moderate, or strong. The ratings for each study were summed to obtain overall scores. A study was rated strong when there were no weak ratings for any of the listed components. Studies with one weak rating were classified as moderate, while those with two or more weak ratings were rated as weak.

### 2.4. Data Synthesis

Meta-analysis was initially planned for this study. However, it could not be performed due to, the heterogeneity of study designs, interventions, reporting, measures and outcomes. Hence narrative synthesis was used to analyse and describe the data. Each included study was summarised by variables such as study design, setting and population, intervention characteristics including duration, drop-out and follow-up, intervention outcomes and measures, and theoretical basis of interventions. Where results were reported for multiple follow-up points during the intervention, only the final results are presented in this review. Intervention effects are presented as mean differences, Cohen’s d, adjusted beta-estimates, only for those primary studies that reported these. Where applicable, simple statistics were computed for changes in outcome variables from baseline to post-intervention and follow-ups and presented as mean differences. The results are grouped and presented according to the outcome measures.

### 2.5. Ethics Consideration

Since this study did not involve the collection of primary data, ethics was not a requirement.

## 3. Results

### 3.1. Description of the Included Studies

**Figure 1:**
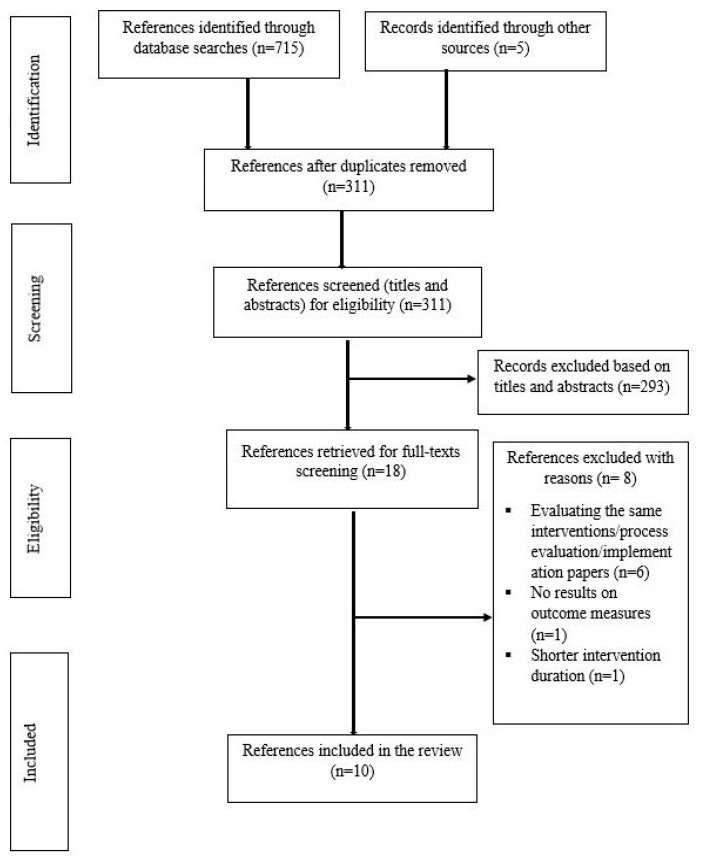
Flow chart for the literature search for intervention studies.

The flowchart for the selection of studies is presented in Figure 1. The database and other searches identified 720 studies. After removal of duplicates, 311 titles and abstracts were screened for eligibility. Eighteen full text articles were reviewed and 10 studies that met the inclusion criteria were retained in this review. Two of these studies evaluated the same intervention [24,25].

#### 3.1.1. Study Setting, Design, and Population

Table 1 shows the characteristics of the included studies. This systematic review included 10 studies conducted among 9957 children and adolescents in two African countries, namely South Africa [24,25,26,27,28,29] and Tunisia [30,31,32,33]. The included studies were published between 2009 and 2017. Four of the studies were described as randomised control trials (RCTs) [24,25,28,33], five were pre- and post-test quasi-experiments [26,27,30,31,32], and one did not report the design [29]. Two of the studies were pilot studies to evaluate the feasibility of the interventions [26,27]. All the studies were primarily school-based studies, with one study [32] involving the community as a secondary setting. Three studies reported enrolling children from both urban and rural schools [24,25,28]. Two studies [24,25] reported that participants were from low socioeconomic settings (defined as quintile 1 and 2 vs. quintile 3 schools). The majority of the participants were primary school children and adolescents in grades 4 to 6 [24,25,26,27,28], and grades 7, 8, and 9 [31,32]. One study [30] explicitly reported recruiting adolescents from public secondary schools, while another [28] involved adolescents from urban and rural settings. The number of children that participated at baseline ranged from 28 [33] to 4003 [32]. Except for one study that involved only boys [29], all the interventions targeted children of both sexes. Of the studies that reported the mean age of participants, this ranged from 12.4 years [28] to 13.5 years [31].

#### 3.1.2. Intervention Characteristics

The duration of the interventions ranged from four months [27,33] to three years [24,25,32], with four lasting less than one year [26,27,29,33]. Post-intervention follow-ups, which were reported in three studies, ranged between 4 months [27,31] and 1 year [32]. The drop-out rates reported in five studies [26,28,30,31,33] ranged from 0.0% [33] (100.0% completed the intervention) to 30.8% [31]. Three of the ten studies were only PA-based interventions [27,29,33] and seven were multicomponent interventions involving both diet and PA only [24,25,31,32] or diet, PA and other health-promoting behaviours such as tobacco use, and also the school environments [26,28,30].

Although the research teams comprised school personnel such as teachers, school doctors and nurses, medical personnel, and student leader groups, the majority of the intervention activities were mainly facilitated by school teachers with additional training [24,25,26,27,29,30,31]. Programmes were presented as interactive sessions, games and sports, group discussions and exercise [27,28,29,30,31,33]. One study conducted a delayed intervention for controls [32], while, in another, the controls were exposed to a type of intervention, namely, prevention of human immunodeficiency virus (HIV)/sexually transmitted disease [28]. Furthermore, four studies integrated their additional sessions into the existing school curricula [25,26,27,30], while two others [28,33] were implemented as extracurricular activities. Additionally, two of the intervention studies targeted overweight and obese children [31,33], one involved parents or caregivers [28] and two involved the school environments by promoting the increased availability of healthy foods at school/tuck shops [26] and provision of PA equipment [31].

#### 3.1.3. Intervention Outcomes and Measures

Two studies [24,27] evaluated PA-related outcomes only, three anthropometric outcomes only [29,31,33], two nutrition- and PA-related outcomes [26,28,30], one nutrition and anthropometric outcomes [25], two nutrition, PA, and anthropometric outcomes [32].

#### 3.1.4. Theoretical Basis of Intervention

The majority of the interventions were not theory based, except for three studies from South Africa. Socioecological theory guided the development of two [24,25], while one was based on social cognitive theory and the theory of planned behaviour [28].

### 3.2. Methodological Quality of the Included Studies

The overall methodological quality of the included studies is presented in Table 1 (details can be found in the online supplementary Table S3). Nine out of the ten studies were categorised as weak and one of high quality [28]. The weak ratings were mainly due to missing information; the authors did not describe the components under consideration in most instances. For example, five studies each were rated either weak or moderate based on selection bias and only one of the four randomised controlled studies described method of randomisation [28]. The drop-out rates, reported by five studies [26,28,30,31,33] were between nil and 30.8%. Information on blinding of assessors to the allocation of treatments in the RCTs and confounding were mostly missing in the studies.

### 3.3. Main Findings of Interventions

#### 3.3.1. Weight Status

The main findings of the interventions are presented in Table 2. Six studies evaluated changes in body composition [25,29,30,31,32,33] and the results were mixed. Of these, three reported statistically significant effects in favour of the intervention groups [31,32,33], while observed changes were not significant in the other two studies [25,29]. It should be noted that two [31,33] of the studies targeted overweight/obese children. Regaieg and co-workers [33] reported a statistically significantly decrease in BMI of children in the intervention group compared to those in the control group (−0.6 kg/m^2^, *p* < 0.001). In addition to targeting obese children, this was a small sample size. Maatoug and colleagues [31] reported a statistically significant decrease in BMI z-score in the post-intervention and follow-up (−0.13, *p* < 0.001 and −0.34, *p* < 0.001 respectively) in the overweight/obese children exposed to the intervention compared to those in the control group. In another study [32], overweight prevalence was significantly reduced in the intervention group (−2.0%, *p* = 0.036) but not the controls (−1.0%, *p* = 0.602). On the contrary, two studies did not observe beneficial effects of the intervention on weight status [25,29].

#### 3.3.2. Physical Fitness, and PA Knowledge, Attitudes, Intentions and Behaviours

Six studies evaluated physical fitness, PA knowledge, attitudes, and behaviours [24,26,27,28,32]. In one study, [26], PA and sports participation increased significantly. This study did not have a control group; however, the number of sports activities that children participated in for at least five times per week increased from 35% to 55% after the intervention (*p* < 0.05). In other studies, more children met the recommended PA guidelines [28,32]. In the study by Jemmott and colleagues [28] for instance, the intervention resulted in significantly more participants meeting PA guidelines in the past seven days compared with controls (odds ratio = 1.56 ((95% confidence interval (CI): 1.29, 1.89)). Ghamman and colleagues [32] reported beneficial effects of the intervention in boys (*p* = 0.021) and older children (*p* = 0.004). For the studies that measured physical fitness, no overall effects were observed on the scores [24,27]. PA self-efficacy [27], knowledge, attitudes and intention improved significantly in children in the intervention but not in the controls [28].

#### 3.3.3. Nutrition Knowledge, Attitudes, Self-Efficacy and Behaviours

Five studies assessed changes in nutrition knowledge, attitudes, self-efficacy and intentions scores, and dietary behaviours including fruit and vegetable intake, fast food intake, and consumption of carbonated drinks [25,26,28,30,32]. Two studies reported statistically significantly increase in the number of participants in the intervention group that met the recommended intake of fruits and vegetables compared with those in the controls. Jemmot et al. [28] reported that children in the intervention group were 1.30 times more likely to meet the recommended intake of fruits and vegetables compared to those in the controls (95% CI: 1.07, 1.58. Likewise, Ghammam [32] found that more children in the intervention groups (+3.2%, *p* = 0.026) met the recommended intake of fruits and vegetables compared with the those in the controls (−5.5%, *p* = 0.001). No significant effects were observed in one study [30]. Moreover, De Villiers [25] did not observe overall significant effects on dietary behaviour. Furthermore, significant improvements were reported in nutrition knowledge, self-efficacy, attitudes and intention in the intervention groups in other studies [25,28,30].

## 4. Discussion

The present systematic review aimed to summarise the available evidence on school-based interventions to prevent childhood overweight/obesity within the African context. A total of ten studies were evaluated. These studies were generally of low methodological quality. The majority of the studies focused on nutrition and physical activity, while a few targeted body composition indices. Moreover, the programme development of the majority of these interventions were not theory based. The results of the effectiveness of these interventions were inconsistent: three of five studies that evaluated weight status, three of six that reported physical activity outcomes, and four of six reporting on nutrition-related outcomes found beneficial effects of the interventions. We are however unable to make definite statements about the overall effectiveness and quality of evidence due to the limited number and heterogeneous outcomes across studies.

These findings highlight the paucity of high-quality, theory based interventions to mitigate the effects of overweight and obesity, and energy-related behaviours among African children. A considerable body of evidence suggests that multicomponent school-based interventions that target PA, dietary behaviours, sedentary behaviours, and the environments are more likely to be effective in children and adolescents compared with single component interventions [15,16,17,18,34]. Given the multi-faceted nature of overweight and obesity, it is not surprising that programmes that target individual behaviours and the obesogenic environments simultaneously are promising. Additionally, these behaviours tend to cluster so that any successful intervention should consider both ends of the energy balance equation. Contrary to the aforementioned studies, the present study found inconsistent results from the multicomponent programmes. It is worth noting that of the single component interventions, positive effects were reported in a small sample of children who were overweight and obese which may not have enough power.

The importance of theoretical frameworks in childhood obesity interventions have been highlighted [35]. In the present review, only three of the ten included studies were theory-based interventions. While one reported beneficial effects in favour of the intervention groups in all the measured outcomes, results from the other two were inconsistent. Some beneficial effects were equally reported in the studies that did not apply theories. Documented evidence indicates that duration of interventions may have an overall impact on adiposity, dietary, and PA behaviours [17,18,19]. Kamath et al. [19] showed that intervention trials with longer duration (>6 months) and post-intervention outcomes tended to yield marginally larger effects. Results from a systematic review [18] showed that studies that reported significant effects were implemented over a longer period compared to those that did not report significant effects. Waters et al. [17] reported that interventions that lasted longer significantly decreased the prevalence of overweight/obesity in preschool children and children aged 6–12 years. The results from the present study are inconsistent; of the studies that lasted >1 year, four yielded statistically significant intervention outcomes, while two were not significant. 

The majority of these interventions were designed to improve dietary and PA activity behaviours by targeting the children. The limited successes of many well-intended behavioural interventions have been attributed to changing behaviours without corresponding changes in the obesogenic environments such as the home, school policies and programmes, advertising, and the community. For school children, the family or home environment is one key setting to target for successful and sustainable interventions. Parents play an influential role in promoting healthy dietary and physical activity behaviours of the children by not only parental practices and rules, but also by providing the supportive environment for these behaviours as well as serving as positive role models [36,37,38]. Parental involvement in school-based health interventions in developed countries is well documented [14,39,40,41]; however, in Africa, there is a paucity of published studies. While there has been considerable interest in parental involvement in school-based obesity interventions, the evidence for programme effectiveness is unclear. Results from one systematic review were inconclusive [39]; however, other studies found positive effects with parental engagements [40,41]. In the present review, one study attempted to involve parents by organising meetings but the turnout was [25]. Another study reported parental involvement through a homework approach [28]. Although this study was effective, no conclusions could be made based on the available evidence.

The results of the present systematic review should be interpreted cautiously. The paucity of studies in Africa is a major limitation; all the studies included in this systematic review were conducted in two African countries and hence the findings could not be generalised to the entire continent. It is possible some relevant studies that were not indexed in the targeted databases were missed in the review process. However, efforts were made to contact key experts across and outside Africa for the existence of relevant studies among African schoolchildren. Additionally, multiple reviewers were involved in the review and interpretation of the results. The methodological quality of the studies was low. Generally, important information such as selection bias, confounding, blinding, reliability, and validity of data collection tools were either missing or not clearly reported. Two of the included studies, however, made references to implementation details elsewhere [24,25]. It should be noted that none of the studies reported adverse effects of the interventions. Moreover, except for body composition and physical fitness, all outcomes relied on self-reports. Reliance on self-reports by children may be subject to recall bias and social desirability, thereby affecting the reliability and accuracy. Furthermore, given that most of the included studies were non-randomised, blinding of participants, data collectors and intervention assessors was not possible. In addition, two studies reported a high attrition rate. Those in the intervention have a high probability of completing the study and may have contributed to the reported effectiveness in the interventions. Meta-analysis was not possible given that the included studies were heterogeneous.

Despite these limitations, this is one of the first systematic reviews of the literature of school-based interventions in the African context. A further strength was the use of the “Effective Public Health Practice Project quality assessment tool for quantitative studies” to assess the quality. While the effectiveness and the evidence from this systematic review may be limited, these broadly agree with the available literature [14,18,19]. The result of this study has research and public health implications. Given the increasing trends of obesity in African children and the limited studies on prevention efforts in the school settings, this study demonstrates the need for further intervention efforts across African countries. There is a need to explore the possibility of rigorous, large, multi-site, well-designed, and theory-driven interventions, with harmonised methodologies and parental involvement. Furthermore, researchers should consider incorporating formative research prior to implementation, as well as integrating interventions into already existing healthy lifestyle school programmes (regular school curricula) and structures to ensure maximum reach, sustainability and effectiveness. Fortunately, in line with the recommendations by World Health Organization (WHO) [42], many African countries have detailed policy initiatives spanning the family, school, the community, and the food and beverage industry. Some of the initiatives targeting educational settings include the creation of healthy food environments in schools and child-care settings by restricting marketing of unhealthy foods and beverages. Additionally, these settings are to provide adequate facilities on school premises and in public spaces for PA during recreational time for all children. It is expected that governments provide the needed resources for the implementation and evaluation of these policy interventions across Africa.

## 5. Conclusions

Overweight and obesity are emerging public health issues among African school children. Given the dearth of studies on school-based obesity interventions and the inconsistent results, definite conclusions about the overall effectiveness could not be made. This study has identified research gaps in the childhood obesity literature in Africa and strengthened the need for further studies. Future studies should focus on objective measures of body composition in addition to targeted energy related behaviours. The findings of such interventions would contribute valuable data which will inform policy.

## Figures and Tables

**Table 1:** Summary characteristics of the included studies on school-based interventions targeting nutrition, physical activity, and weight status of children in African countries.

Reference	Design, Setting and Population	Intervention Characteristics		Intervention Outcomes			Measures	Theoretical Basis	Overall Quality
		Intervention Components	Duration, Follow-Up and Drop-Outs	Weight Status	Nutrition	PA			
Naidoo et al., 2009 [26]	Design: Cohort (one group pre- and post-test) Setting: Four primary schools in KwaZulu- Natal, South Africa Participants: 256 children in grade 6 from low- to middle- income settings. Boys/girls: 81/104	Intervention: Diet, PA and school environment Concepts of PA and healthy eating habits were integrated with the existing curriculum. Programme was implemented by school personnel. Teachers were to advise and prompt children to make healthy choices. Schools were to establish health promoting environments by increasing the availability of healthy foods and decrease unhealthy foods at school/tuck shops.	Duration: 6 months Drop-outs: 71 (27.7%)	√		√	BMI, increased participation in sports and PA and the availability of healthy food choices.	No	Weak
Draper et al., 2010 [27]	Design: Pre- and post-test study Setting: Five elementary/primary schools in Alexandra Township, Gauteng Province, South Africa Participants: 508 children in grade 4–6 Age: NR Boys/girls: NR	Intervention: PA Teachers provided physical education as part of an integrated curriculum (Healthnutz project) to children, while situational analysis and focus group discussions were conducted for teachers and research team monitors.	Duration: 6 months Follow-up: 4 months	√		√	Physical fitness, knowledge, self-efficacy and attitudes and weight.	No	Weak
Harrabi et al., 2010 [30]	Design: Pre- and post-test quasi experiment Setting: Seventy-six classes from four public secondary schools in Tunisia Participants: 2200 children Age (range): 12–16 years Boys/girls: 1026/1174	Intervention: Diet, PA and tobacco use This was delivered by project team, teachers and school doctors. Cognitive behavioural components of health knowledge and health promoting concepts such as tobacco use, PA, and healthy diet were integrated with the biological sciences and physical education curriculum.	Duration: 1 year Drop-outs: 138 (5.9%)		√	√	Knowledge, intentions, behaviours of PA and nutrition.	No	Weak
Jemmott et al., 2011 [28]	Design: RCT Setting: 18 schools (14 urban and four rural) in Eastern Cape Province, South Africa Participants: 1057 children in grade 6 Age (mean): 12.4 years; 9–17 years Boys/girls: 558/499	Intervention Diet, PA and cognitive-behavioural health Consisted of 12 one-hour modules, with two modules delivered during each of the six sessions on six consecutive school days; extracurricular sessions held at the end of the school day and included interactive exercises, games, brainstorming, role-playing and group discussions. Homework approach to involve parents or caregivers.	Duration: 13 months Drop-outs: 35 (3.3%)		√	√	Nutrition and PA knowledge, attitudes, self-efficacy and behaviours.	Social cognitive theory and the theory of planned behaviour	Strong
Monyeki et al., 2012 [29]	Design: NR Setting: Two primary schools in Gauteng Province, South Africa Participants: 322 children Age (range): 9–13 years Boys: 322	Intervention: PA Two 30 min exercise sessions per week during school hours. Lessons consisted of warm-up with stretching exercises, speed, strength, balance and cool down exercises. Intervention was provided by a trained physical education teacher.	Duration: 10 mo	√			BMI and body fat.	No	Weak
Regaieg et al., 2013 [33]	Design: RCT Setting: Elementary schools in Sfax, Tunisia. Participants: 28 obese children Age (range): 12–14 years Boys/girls:16/12	Intervention: PA Four extracurricular sessions (two sessions on weekdays and two on weekends) of 60 min per week aerobic exercises in addition to regular physical education that was provided by the schools. Exercises were performed under the supervision of a cardiologist.	Duration: 4 months Drop-outs: 0%	√			BMI, weight, waist circumference and FFM.	No	Weak
Maatoug et al., 2015 [31]	Design: Quasi-experiment Settings: Six schools in Sousse, Tunisia Participants: 585 obese and overweight children in grades 7 and 8 Age: 13.1 ± 0.9 y and 13.5 ± 0.9 years in intervention and control groups Boys/girls: 236/349	Intervention: Diet and PA School personnel including PA teachers and parents were trained on the relevance of healthy behaviours in obesity management. Schools were provided with PA equipment. Children were motivated to engage in regular PA and follow healthy diets in collective interactive sessions twice a week, with each session lasting one hour, as well as individual sessions for obese children. Intervention was facilitated by a dietician, psychologist, medical doctor and teachers (“Contrepoids” program).	Duration: 1 year Follow-up: 4 months Drop-outs: 180 (30.8%)	√			BMI and zBMI.	No	Weak
De Villiers et al., 2016 [25] ^a^	Design: Cluster RCT Setting: 16 primary schools (eight urban and eight rural) in low SES settings in Western Cape, South Africa Participants: 998 children in grade 4 Boys/girls: 471/526	Intervention: Diet and PA HealthKick activities included the improvement of the school nutrition environment by developing healthy school nutrition policies, promoting the availability of healthier food options, initiation of vegetable gardens at schools and providing nutrition education support. Teachers were given training and resources, and were to organise an additional 15 min of PA per day and at least one healthy eating activity per month. Intervention was integrated with the existing nutrition curriculum.	Duration: 3 years	√	√		Nutrition behaviour, self-efficacy, overweight and obesity.	Socioecological theory	Weak
Uys et al., 2016 [24] ^a^	Design: Cluster RCT Setting: 16 primary schools (eight urban and eight rural) in low SES settings Participants: 998 children in grade 4 Boys/girls: 471/526	Intervention: Diet and PA This was implemented by the intervention schools that were also given a toolkit containing teachers’ manual, curriculum manual, a resource box and PA resource bin (HealthKick).	Duration: 3 years			√	Physical fitness levels and PA-related knowledge, attitudes and behaviours.	Socioecological theory	Weak
Ghamman et al. 2017 [32]	Design: Quasi-experiment Setting: 17 schools in Sousse, Tunisia Participants: 4003 children in grades 7 and 9 Age: 11–16 years Boys/girls: 1933/2070	Intervention: Diet and PA Educational events were organised at least three times in a school year for children, parents and teachers. Classroom sessions were organised by teachers and consisted of interactive lessons of healthy eating, the benefits of regular PA, and ways to incorporate PA into usual activities. After-school soccer games were organised both within and between the schools to encourage PA. Programmes were delivered by student leaders, project team and teachers (“Together in Health”).	Duration: 3 years Follow-up: 1 year	√	√	√	Weight status, PA, screen time behaviours, fruit and vegetable intake and fast food intake.	No	Weak

NR: not reported; RCT: randomised controlled trial; BMI: body mass index; BMI z-scores: body mass index z-scores; PA; physical activity; ^a^ these two studies evaluated the same intervention (HealthKick) with different outcomes measures; SES: socioeconomic status (defined as quintile 1 and 2 vs. quintile 3 schools); RCT: randomised control trial.

**Table 2:** Summary of results of school-based interventions targeting nutrition and physical activity, and body weight status of children in African countries.

Reference and Outcome	Change Over Time in I and C and I vs. C					Intervention Effects as Reported in Primary Studies	Main Findings
	∆I	∆C	*p*-Value	∆I–∆C	*p*-Value		
Naidoo et al., 2009 [26] ^†^							PA and sports participation increased significantly post-intervention (*p* < 0.05). Healthy food and drinks choices were available.
Number of sports participated in (average)	10.0 *						
PA > 5 times/week after school (%)	20.0 *						
Boys							
Sit ups	+2.0						
Sit and reach (cm)	+0.29						
Standing broad jump (m)	+1.0						
BMI (kg/m^2^)	+0.8						
Girls							
Sit ups	+1.0						
Sit and reach (cm)	+0.89						
Standing broad jump (m)	+0.0						
BMI (kg/m^2^)	+0.65						
Draper et al., 2010 [27]							Intervention improved self-efficacy for PA in the experimental group but not the controls (*p* < 0.05). PA knowledge improved in both the intervention and control groups. There was no effect on overall physical fitness scores. However, significant effects on sit and reach (*p* < 0.001), sit ups (*p* < 0.02), and shuttle run (*p* < 0.0001) between intervention and control groups were reported. Weight of children in the intervention significantly decreased, while change was reported for height.
Sit and reach (cm)	+4.40	−10.50	<0.001				
Sit ups (in 30 s)	+1.80	+0.30	<0.02				
Shuttle run (seconds)	−2.30	+1.40	<0.0001				
Long jump (cm)	+9.70	+14.6	NS				
Ball throw (m)	−1.10	+0.10	NS				
PA self-efficacy	+0.30	−0.01	<0.05				
PA knowledge	+0.56	+0.47	NS				
Harrabi et al., 2010 [30]							Nutrition knowledge and intention improved significantly in the intervention compared to the control group. The percentage of children with increased intake of fruits and vegetables increased in both groups, although significant in the controls. PA intention (*p* < 0.001) and behaviour (*p* < 0.001) improved in the intervention group. No significant differences in BMI in both groups.
What to eat for breakfast (%)	+25.1	+1.2		+22.9	0.0001		
Intention to eat breakfast (%)	+8.2	+2.9		+7.3	0.0001		
Fruit and vegetable intake ≥ 5 times/day (%)	+10.1	+9.6		−2.5	NS		
Intention to engage in PA daily (%)	+9.1	+1.7		+3.5			
PA duration ≥ 30 min for at least six days a week (%)	+18.4	+9.7		−1.0	0.0001		
Jemmott et al., 2011 ^a^ [28]							More participants in health-promotion intervention than controls met 5-a-Day fruit (*p* = 0.003) and vegetable (*p* = 0.0001) intake, and PA guidelines (*p* = 0.0001). Health-promotion knowledge, attitude and intention increased (all *p* < 0.0001) in the intervention group.
Fruit and vegetable intake ≥ 5 times/day in the past 30 days (5-a-Day) (%)	+2.83	−5.70			0.008	+0.16	
Mean servings of fruit per day in the past 30 days	+0.49	+0.33			0.003	+0.19	
Mean servings of vegetables/day in the past 30 days	+0.98	+0.17			0.0001	+0.24	
Meeting PA guidelines in the past 7 days (%)	+7.10	+7.10			0.0001	+0.27	
Health knowledge	+3.48	+1.38			0.0001	+1.03	
Attitude toward health-promoting behaviour	+1.14	+0.69			0.0001	+0.89	
Intention for health-promoting behaviour	+1.02	+0.54			0.0001	+0.81	
Monyeki et al., 2012 [29]							Non-significant decreasing trends in BMI and percentage body fat (*p* = 0.32) in intervention group, whereas BMI tended to be stable with an increasing percentage body fat by age in the control group.
Body fat at age 12 y (%)	−0.32	+1.62	NS				
Body fat at age 13 y (%)	−1.03	+2.31	NS				
Regaieg et al., 2013 [33]							Significant decreases in BMI, FM and waist circumference in intervention (*p* < 0.001). In the controls, a non-significant increase (*p* = 0.11) in waist circumference was observed. There were increases in FFM in both groups, but this was higher in the intervention.
Weight (kg)	+0.70	+2.60	<0.001				
BMI (kg/m^2^)	−0.60	+0.50	<0.01				
FM (%)	−4.30	−0.20	<0.01				
Waist circumference (cm)	−1.70	+0.70	<0.001				
Maatoug et al., 2015 [31]							BMI z-score decreased significantly from pre-intervention to post-intervention and from post-intervention to 4-mo follow-up in the intervention group. In the control group, BMI z-score decreased significantly from pre- to post- intervention but nor from post- to follow-up.
BMI (kg/m^2^)	+0.25	+0.49 ***					
BMI z score	−0.13 ***	−0.18 ***					
De Villiers et al., 2016 ^a^ [25]							Nutrition knowledge (*p* = 0.011) and self-efficacy (*p* = 0.039) significantly improved in the intervention group as compared with the controls. The intervention did not improve nutrition behaviour (*p* = 0.743) nor weight status of the children.
Nutrition knowledge	+2.52	+0.60				+1.92 **	
Nutrition behaviour	−0.52	−0.60			NS	+0.09	
Self-efficacy	+0.36	−0.35				+0.71 *	
Overweight (%)	+1.00	+1.00					
Obesity (%)	−4.00	+7.00					
Uys et al., 2016 ^a^ [24]							Intervention did not improve overall physical fitness and determinants of PA behaviour. PA knowledge improved in both intervention (*p* < 0.005) and control ((*p* < 0.001) groups. Additionally, improvement was only observed in the sit-ups score of children in the intervention group (*p* < 0.05).
PA knowledge						−0.48 *	
PA behaviour						−0.44	
PA self-efficacy						−0.38	
Sit and reach (cm)						−1.29	
Sit ups (in 30 s)						+1.62 *	
Shuttle run (seconds)						+3.32	
Long jump (cm)						−5.75	
Ghamman et al., 2017 [32]							Overall, higher proportion of children (*p* = 0.010), boys (*p* = 0.021) and those ≥ 14 years (*p* = 0.004) in the intervention group met the recommended daily PA post-intervention, whereas, in the controls, an increase was observed only at follow-up (*p* = 0.023). Further, more children in the intervention group reported eating five fruits and vegetables daily (*p* = 0.02). Overweight prevalence reduced in the intervention group (*p* = 0.036).
Fruit and vegetable intake ≥ 5 times/day (%)	+3.2 *	−5.2 **					
Fast food consumption ≥ 4 times/week (%)	−0.8	+5.1 ***					
Meeting recommended PA (%)	−3.6 *	+0.1					
Weekday screen time > 2 hr/day (%)	+1.4	−2.1					
Weekend screen day > 2 hr/day	−0.1	−7.0 ***					
Prevalence of overweight (%)	−2.6 *	−1.0					

NS: not significant; *** *p* < 0.001; ** *p* < 0.01; * *p* < 0.05; ^a^: adjusted beta estimates for baseline prevalence; FM: fat mass; FFM: fat free mass; PA: physical activity; BMI: body mass index; BMI z-score: body mass index z-score; hr: hour; I: intervention; C: control; IE: intervention effects; ∆I: change in intervention; ∆C: change in control; ∆I–∆C: difference between change in intervention and control; ^†^: no controls, change in intervention are presented by gender.

## Supplementary Materials

The following are available online at https://www.mdpi.com/2072-6643/12/1/95/s1. Table S1: Search Strategy PubMed; Table S2: PRISMA checklist; Table S3: Quality of included studies.
