# Supplementary material for: School-Based Interventions Targeting Nutrition and Physical Activity, and Body Weight Status of African Children: A Systematic Review
Source: Nutrients. 2019 Dec 30;12(1):95. doi: 10.3390/nu12010095 (PMC7019429; doi:10.3390/nu12010095)
Supplement: Supplementary file 1 [file nutrients-12-00095-s001.pdf]

Supplementary material

School-Based Interventions Targeting Nutrition and Physical Activity Behaviours, and Body Weight Status of African Children: A Systematic Review

Theodosia Adom [1, 2,*], Anniza De Villiers [3], Thandi Puoane [1] and André Pascal Kengne [4]

Table S1: PRISMA checklist

Section/topic	#	Checklist item	Reported on page #
**TITLE**			
Title	1	Identify the report as a systematic review, meta-analysis, or both.	1
**ABSTRACT**			
Structured summary	2	Provide a structured summary including, as applicable: background; objectives; data sources; study eligibility criteria, participants, and interventions; study appraisal and synthesis methods; results; limitations; conclusions and implications of key findings; systematic review registration number.	1
**INTRODUCTION**			
Rationale	3	Describe the rationale for the review in the context of what is already known.	2
Objectives	4	Provide an explicit statement of questions being addressed with reference to participants, interventions, comparisons, outcomes, and study design (PICOS).	2
**METHODS**			
Protocol and registration	5	Indicate if a review protocol exists, if and where it can be accessed (e.g., Web address), and, if available, provide registration information including registration number.	2, PROSPERO, # CRD42016035248
Eligibility criteria	6	Specify study characteristics (e.g., PICOS, length of follow-up) and report characteristics (e.g., years considered, language, publication status) used as criteria for eligibility, giving rationale.	2 & 3
Information sources	7	Describe all information sources (e.g., databases with dates of coverage, contact with study authors to identify additional studies) in the search and date last searched.	3
Search	8	Present full electronic search strategy for at least one database, including any limits used, such that it could be repeated.	3, Table S1
Study selection	9	State the process for selecting studies (i.e., screening, eligibility, included in systematic review, and, if applicable, included in the meta-analysis).	3
Data collection process	10	Describe method of data extraction from reports (e.g., piloted forms, independently, in duplicate) and any processes for obtaining and confirming data from investigators.	3
Data items	11	List and define all variables for which data were sought (e.g., PICOS, funding sources) and any assumptions and simplifications made.	3
Risk of bias in individual studies	12	Describe methods used for assessing risk of bias of individual studies (including specification of whether this was done at the study or outcome level), and how this information is to be used in any data synthesis.	3
Summary measures	13	State the principal summary measures (e.g., risk ratio, difference in means).	3 & 4
Synthesis of results	14	Describe the methods of handling data and combining results of studies, if done, including measures of consistency (e.g., I^2) for each meta-analysis.	3 & 4

Table S2: Search strategy PubMed: Protocol for systematic review of school-based interventions to prevent and control obesity in African learners

Search	Search terms	Hits
1	Weight [tw] OR height [tw] OR BMI [tw] OR BMI z-scores [tw]	
2	Obesity, Overweight [MeSH Terms]	
3	Obesity prevention [tw] OR obesity treatment [tw] OR obesity management [tw] OR health promotion [tw] OR health education [tw] OR physical activity [tw] recreation* [tw] OR sports [tw] OR exerci* [tw] OR fitness [tw] OR nutrition intervention [tw] OR diet* intervention [tw]	
4	School programme [tw] OR school intervention [tw] OR school-based study [tw]	
5	# 1 OR # 2 OR # 3 OR # 4	
6	Learner* [tw] OR school* children [tw] OR school* going children [tw]	
7	# 5 AND # 6	
8	((((("Africa"[MeSH] OR Africa*[tw] OR Algeria[tw] OR Angola[tw] OR Benin[tw] OR Botswana[tw] OR "Burkina Faso"[tw] OR Burundi[tw] OR Cameroon[tw] OR "Canary Islands"[tw] OR "Cape Verde"[tw] OR "Central African Republic"[tw] OR Chad[tw] OR Comoros[tw] OR Congo[tw] OR "Democratic Republic of Congo"[tw] OR Djibouti[tw] OR Egypt[tw] OR "Equatorial Guinea"[tw] OR Eritrea[tw] OR Ethiopia[tw] OR Gabon[tw] OR Gambia[tw] OR Ghana[tw] OR Guinea[tw] OR "Guinea Bissau"[tw] OR "Ivory Coast"[tw] OR "Cote d'Ivoire"[tw] OR Jamahiriya[tw] OR Jamahiryia[tw] OR Kenya[tw] OR Lesotho[tw] OR Liberia[tw] OR Libya[tw] OR Libia[tw] OR Madagascar[tw] OR Malawi[tw] OR Mali[tw] OR Mauritania[tw] OR Mauritius[tw] OR Mayote[tw] OR Morocco[tw] OR Mozambique[tw] OR Mocambique[tw] OR Namibia[tw] OR Niger[tw] OR Nigeria[tw] OR Principe[tw] OR Reunion[tw] OR Rwanda[tw] OR "Sao Tome"[tw] OR Senegal[tw] OR Seychelles[tw] OR "Sierra Leone"[tw] OR Somalia[tw] OR "South Africa"[tw] OR "St Helena"[tw] OR Sudan[tw] OR Swaziland[tw] OR Tanzania[tw] OR Togo[tw] OR Tunisia[tw] OR Uganda[tw] OR "Western Sahara"[tw] OR Zaire[tw] OR Zambia[tw] OR Zimbabwe[tw] OR "Central Africa"[tw] OR "Central African"[tw] OR "West Africa"[tw] OR "West African"[tw] OR "Western Africa"[tw] OR "Western African"[tw] OR "East Africa"[tw] OR "East African"[tw] OR "Eastern Africa"[tw] OR "Eastern African"[tw] OR "North Africa"[tw] OR "North African"[tw] OR "Northern Africa"[tw] OR "Northern African"[tw] OR "South African"[tw] OR "Southern Africa"[tw] OR "Southern African"[tw] OR "sub Saharan Africa"[tw] OR "sub Saharan African"[tw] OR "subSaharan Africa"[tw] OR "subSaharan African"[tw]) NOT ("guinea pig"[tw] OR "guinea pigs"[tw] OR "aspergillus niger"[tw])))))	
9	# 7 AND # 8	
10	# 9 Limits: 2000/01/01 to 2018/06/30	

Table S3: Quality of included studies

Reference	Selection bias	Study design	Confounders	Blinding	Data collection methods	Withdrawals and drop-outs	Overall rating
	Likelihood of bias due to the allocation process in an experimental study Participants are more likely to be representative of the target population	*Was the study described as randomized? Was the method of randomization described?*	*The authors should indicate if confounders were controlled in the design (by stratification or matching) or in the analysis. If the allocation to intervention and control groups is randomized, the authors must report that the groups were balanced at baseline with respect to confounders.*	*Assessors should be described as blinded to which participants were in the control and intervention groups. Study participants should not be aware of (i.e. blinded to) the research question*	*Tools for primary outcome measures must be described as reliable and valid*	*Numbers and reasons for withdrawals and drop-outs must be described*	
Naidoo et al, 2009 [19]	Weak	Moderate	Weak	Weak	Moderate	Moderate	Weak
Draper et al, 2010 [20]	Weak	Moderate	Weak	Weak	Moderate	Weak	Weak
Harrabi et al, 2010 [22]	Moderate	Moderate	Weak	Weak	Moderate	Strong	Weak
Jemmott et al, 2011 [21]	Moderate	Strong	Strong	Strong	Strong	Strong	Strong
Monyeki 2012 [15]	Weak	Weak	Weak	Weak	Moderate	Weak	Weak
Regaieg 2013 [25]	Weak	Strong	Strong	Weak	Moderate	Strong	Weak
Maatoug et al, 2015 [23]	Weak	Moderate	Weak	Weak	Moderate	Moderate	Weak
De Villiers et al, 2016 [18]	Moderate	Strong	Weak	Moderate	Moderate	Weak	Weak
Uys 2016 [17]	Moderate	Strong	Moderate	Weak	Strong	Weak	Weak
Ghamman 2017 [24]	Moderate	Moderate	Weak	Weak	Strong	Weak	Weak
